# Phenolic Profiling and OECD-Guided Acute and Subacute Oral Toxicity Assessment of *Cannabis sativa* L. Seed Hydroethanolic Extract

**DOI:** 10.3390/molecules31203587

**Published:** 2026-10-09

**Authors:** Rafik El-Mernissi, Naoual El Menyiy, Aziz Zouhri, Yahya El-Mernissi, Ghizlane Nouioura, Mohamed Bouhrim, Moneerah J. Alqahtani, Jawaher H. Alqahtani, Joe Miantezila Basilua, Nadia Senhaji, Oualid Abboussi, Lhoussain Hajji

**Affiliations:** 1 Human Nutrition, Bioactives and Oncogenetics Team, Faculty of Sciences, Moulay Ismail University, Meknes 50070, Morocco; 2 Physiology and Physiopathology Team, Faculty of Sciences, Genomic of Human Pathologies Research Centre, Mohammed V University, Rabat 1014, Morocco; 3 Laboratory of Pharmacology, National Agency for Medicinal and Aromatic Plants, Taounate 34025, Morocco; 4 Applied Chemistry Research Unit, Faculty of Science and Techniques, Abdelmalek Essaadi University, Al-Hoceima 32000, Morocco; 5 Laboratory of Drug Sciences, Faculty of Medicine, Pharmacy, and Dental Medicine of Fez, Sidi Mohamed Ben Abdellah University, Fez 30000, Morocco; 6 Biological Engineering Laboratory, Faculty of Sciences and Techniques, Sultan Moulay Slimane University, Beni Mellal 23000, Morocco; 7 Department of Pharmacognosy, College of Pharmacy, King Saud University, P.O. Box 2457, Riyadh 11451, Saudi Arabia; 8 Department of Biostatistics and Mathematics, Faculty of Pharmacy, Paris Cité University, 4, Avenue de l’Observatoire, 75006 Paris, France

**Keywords:** *Cannabis sativa* L. seeds, ethnopharmacology, phenolic profiling, HPLC-DAD, SDG3, safety assessment, OECD guidelines

## Abstract

*Cannabis sativa* L. seeds have been traditionally used in Moroccan folk medicine and are increasingly recognized as a valuable source of nutrients and bioactive phytochemicals with promising therapeutic properties. Despite their widespread traditional and nutritional use, scientific evidence supporting their toxicological safety remains limited. This study aimed to characterize the phenolic profile of a hydroethanolic extract of *Cannabis sativa* L. seeds and to evaluate its acute and 28-day repeated-dose oral toxicity in mice following the Organization for Economic Cooperation and Development (OECD) guidelines. The phenolic composition of the extract was analyzed using high-performance liquid chromatography coupled with diode-array detection (HPLC-DAD). Acute oral toxicity was assessed according to OECD Guideline 423 after a single oral administration of 5000 mg/kg body weight. Repeated-dose oral toxicity was evaluated following OECD Guideline 407 by administering the extract orally at doses of 500 and 1000 mg/kg/day for 28 consecutive days. Clinical observations, body weight, food and water intake, relative organ weights, hematological and biochemical parameters, and histopathological examinations of the liver and kidneys were performed. HPLC-DAD analysis identified seven major phenolic compounds, namely catechin, chlorogenic acid, caffeic acid, vanillin, *p*-coumaric acid, quercetin, and kaempferol. No mortality, behavioral abnormalities, or treatment-related clinical signs were observed during either the acute or repeated-dose studies. The extract produced no significant alterations in body weight gain, food and water consumption, relative organ weights, liver and kidney histology, or most hematological and biochemical parameters. A significant reduction in serum total cholesterol and triglyceride levels was observed in treated animals, while platelet counts were significantly reduced only in female mice receiving 1000 mg/kg/day. Under the experimental conditions employed, the hydroethanolic extract of *Cannabis sativa* L. seeds exhibited a favorable oral safety profile, with an estimated LD_50_ greater than 5000 mg/kg. These findings support the traditional use and potential nutraceutical applications of *Cannabis sativa* seeds and provide a scientific basis for further pharmacological investigations and long-term safety studies.

## 1. Introduction

*Cannabis sativa* L., commonly known as hemp, has been cultivated and used across diverse cultures for millennia, valued primarily for its fiber and, more recently, for its medicinal and nutritional properties [1,2]. In Morocco, particularly in the Rif region where the plant has long been cultivated, the seeds have traditionally been regarded as a by-product of hemp cultivation and used mainly as bird feed and animal fodder or pressed to obtain oil used in dermatological applications, rather than for direct human nutritional consumption [2,3,4]. This historical perception of the seeds as a low-value residue has, until recently, limited their scientific and therapeutic exploitation compared to other parts of the plant. However, growing evidence of their rich phytochemical and nutritional composition has shifted this perspective, positioning *C. sativa* seeds as an underexploited source of bioactive compounds worthy of pharmacological and toxicological investigation [5]. Beyond their traditional use, these seeds are increasingly recognized for their rich nutritional and phytochemical profile, combining fatty acids, proteins, and a diverse array of polyphenolic constituents with recognized antioxidant and anti-inflammatory properties [6,7]. This composition supports their proposed therapeutic potential in the management of chronic conditions such as diabetes, inflammatory disorders, and cardiovascular diseases [8]. To effectively harvest these non-polar and polar phytochemicals, hydro-ethanolic systems are preferred over pure organic solvents or water alone. Combining ethanol and water creates a synergistic solvent polarity capable of breaching cell wall barriers, swelling the plant matrix, and dissolving a broad spectrum of both hydrophilic and amphiphilic secondary metabolites [9]. However, despite the promising therapeutic and nutraceutical potential of extracts rich in such complex secondary metabolites, high biological activity does not inherently guarantee safety. Evaluating the innocuity of bioactive-rich extracts is a mandatory prerequisite prior to any clinical or nutraceutical application. While the toxicity of cannabinoid-rich aerial parts is well-documented, systematic safety data for the hydro-ethanolic seed extracts remain strikingly scarce.

To bridge this critical knowledge gap, the present study comprehensively evaluates the phytochemical profile and safety of Moroccan *Cannabis* seed extract using both acute (single dose) and sub-acute (28-day repeated dose) in vivo oral toxicity models [10]. By evaluating clinical signs, hematological parameters, biochemical markers, and histopathological changes, this work establishes baseline safety guidelines for future therapeutic applications.

Ultimately, this research aims to characterize the phenolic composition of Moroccan *C. sativa* L. seed hydroethanolic extract and evaluate its acute and repeated-dose oral toxicity to support its traditional medicinal use, constituting one of the first preclinical toxicological evaluations of these seed extracts conducted in strict accordance with standardized OECD guidelines.

## 2. Materials and Methods

### 2.1. Plant Material and Extract Preparation

Fresh seeds of *C. sativa* L. were collected in September 2021 from the Tafrant region (34°39′28.4″ N, 5°05′58.9″ W) in Taounate, Morocco. The plant specimens were authenticated at the Scientific Institute of Rabat, and a voucher specimen, cataloged as RAB 112735, was preserved in the institute’s herbarium for future reference. The seeds were thoroughly washed three times with pure hexane to remove any residual THC [11]. They were then dried at room temperature until they reached a constant weight, after which they were ground into a fine powder using an electric grinder. A total of 100 g of the powdered seeds were mixed with 1000 mL of a 70% hydro-ethanolic solution and macerated for 48 h at room temperature. After filtration, the filtrate was evaporated using a rotavapor (Büchi R-205 Labortechnik AG, Flawil, Switzerland) at 40 °C, followed by lyophilization. The extraction yield was determined relative to the initial dry plant mass and expressed as a weight-to-weight percentage (% *w*/*w*). It was calculated using the following equation:

$$\mathrm{Extraction}\;\mathrm{yield}\;(\%)=\frac{\mathrm{Weight}\;\mathrm{of}\;\mathrm{dry}\;\mathrm{extract}\;\mathrm{obtained}}{\mathrm{Weight}\;\mathrm{of}\;\mathrm{dry}\;\mathrm{plant}\;\mathrm{material}}\times 100$$

### 2.2. HPLC-DAD Analysis

The phytochemical profile of the seed extract (CSSE) was analyzed using reverse-phase high-performance liquid chromatography coupled with a diode array detector (HPLC-DAD (Thermo Scientific, Thermo Fisher Scientific Inc., Waltham, MA, USA). Prior to injection, the extract and standards were reconstituted at a concentration of 10 mg/mL and filtered through 0.45 μm membrane filters to remove particulate matter, ensuring column protection and analytical reproducibility. Chromatographic separation was performed on a Thermo Scientific HPLC system equipped with a MOS-1 HYPERSIL analytical column (250 × 4.6 mm, 5 μm particle size; SS Exsil ODS), maintained at a constant temperature of 30 °C throughout the analysis. Elution was carried out in gradient mode using a binary mobile phase composed of acidified water (0.1% acetic acid) (solvent A) and acetonitrile (solvent B), according to the following program: 80% A and 20% B for the first minute; followed by 60% A and 40% B for 2.5 min; and returning to 80% A and 20% B for the final 4 min. The mobile phase was delivered at a constant flow rate of 1 mL/min, with an injection volume of 5 μL. Detection was performed over the 200–400 nm spectral range, and chromatographic profiles were recorded at 280 nm, a wavelength selected for its sensitivity to phenolic and flavonoid compounds. The HPLC-DAD analysis was performed for qualitative phytochemical profiling and identification of the detected compounds. Therefore, the chromatographic data were interpreted qualitatively based on retention time and UV spectral comparison with authentic reference standards analyzed under identical [12].

### 2.3. Animals

Swiss mice used in this study, approximately 6 weeks old and weighing between 20 and 23 g, were obtained from the Faculty of Sciences Moulay Ismail Meknes facility. The animals were separated by gender and housed under ethical authorization number 86/609/EC20 in standard conditions. They were kept in standard environmental conditions, with a 12 h light/dark cycle, temperature controlled at 22 ± 3 °C, and 55% relative humidity. Water and food were provided ad libitum, and fresh soft wood splinters were regularly supplied as bedding to prevent health risks and infections, ensuring cleanliness. Every effort was made to minimize animal suffering, distress, and the total number of animals used throughout the experimental protocols.

### 2.4. Safety and Toxicological Assessment of Cannabis sativa L. Seed Extract

#### 2.4.1. Single-Dose Acute Toxicity Study

The acute oral toxicity test was conducted following the directives outlined in the Organization for Economic Co-operation and Development’s Guideline 423 [13] (Figure S1). Animals were randomly allocated to the experimental groups using a simple randomization procedure to minimize selection bias. Twenty mice of both genders were employed in this experiment, haphazardly divided into two groups, each comprising 5 males and 5 females: one designated as the control group (given distilled water 1 mL/100 g), and the other as the dose group (seed extract (CSSE) 5000 mg/kg). Animals were deprived of food overnight but had access to water, and then the extract and distilled water were orally administered. During the initial day, the mice were observed for 4 h, with monitoring intervals every thirty minutes. The animals were subsequently monitored daily for a period of 14 days for indications for signs of acute toxicity, mortality, and behavioral alterations such as aggression, agitation, drowsiness, breathing difficulties, seizures, and paralysis.

#### 2.4.2. Repeated Dose 28-Day Oral Toxicity Study

##### Study Design

Sub-acute toxicity was evaluated by daily exposure of mice to a vehicle (distilled water 1 mL/100 g) or *C. sativa* L. seed extract (CSSE) 500 mg/kg orally or 1000 mg/kg orally) for a duration of 28 days in compliance with OECD guideline 407 [14]. The selected doses were based on the results of the acute oral toxicity study, in which no mortality or treatment-related signs of toxicity were observed following administration of a single oral dose of 5000 mg/kg. In accordance with the recommendations of OECD Guideline 407, which advocates selecting repeated-dose levels based on available acute toxicity data, doses of 500 and 1000 mg/kg (corresponding to 10% and 20% of the highest non-toxic dose, respectively) were considered appropriate to assess the safety of repeated oral administration while avoiding excessive exposure. Thirty mice, encompassing both males and females, were divided into three groups (group 1: control group; group 2: 500 mg/kg of CSSE; group 3: 1000 mg/kg of CSSE), with each group comprising 5 males and 5 females. Throughout the experiment, the mice’s weights were measured weekly, fresh solutions of the extracts were prepared daily before use; the dose was adjusted according to the most recent body weight of each animal, and the corresponding administration volume was calculated individually to maintain the intended dose in mg/kg body weight throughout the treatment period. The animals were continually observed for both signs of illness and mortality; water consumption and food intake were recorded every day.

On the 29th day, following an overnight fast of approximately 12 h with free access to water, all treated mice underwent anesthesia using isoflurane solution; following complete loss of consciousness and pedal reflexes, cervical dislocation was performed, and blood samples were promptly collected to study serum biochemical assays and hematological parameters. The organs, including the liver, spleen, kidneys, and heart, were extracted, cleared of connective tissue, washed with 0.9% NaCl solution, examined visually, and weighed to calculate the organ index using the formula: relative organ index = (organ weight/body weight) × 100. The liver and kidneys were then preserved in 10% formaldehyde for histopathological analysis purposes.

##### Hematological Parameters

Blood samples collected in tubes containing EDTA, were subjected to analysis, utilizing an automatic hematologic analyser (Yumizen H-500 HORIBA Medical, Montpellier, France) for the assessment of the following hematological parameters: White blood cell (WBC), Red blood cell (RBC), Lymphocyte (LYM), Monocyte (MONO), Neutrophil (NEU), Eosinophils (EOS), and Basophils (BAS) Platelet count (PLT), Hemoglobin (HGB), Haematocrit (HCT), Mean corpuscular volume (MCV), mean corpuscular hemoglobin (MCH), Mean corpuscular hemoglobin concentration (MCHC),

##### Biochemical Parameters

Serum samples underwent biochemical analysis conducted through an automated chemistry analyzer ((KONELAB 20i, Thermo Fisher Scientific, Vantaa, Finland). The primary biochemical markers were alanine aminotransferase (ALT), aspartate aminotransferase (AST), creatinine (Cr), triglyceride (TG), total cholesterol (TC), urea (Ur), fasting blood glucose (FBG), and total protein (TP).

##### Histopathological Examination

The liver and kidneys were fixed in a 10% buffered formalin solution. Afterward, they underwent standard histological procedures, where they were encased in paraffin, sliced into sections 6 µm thick, followed by staining with hematoxylin and eosin to facilitate structure identification, and then examined microscopically.

### 2.5. Statistical Analyses

All findings are depicted as the mean value ± standard deviation (SD). Intra-group comparisons were conducted using two-way analysis of variance (ANOVA) with a Tukey post hoc test in GraphPad Prism version 9. The outcomes were deemed statistically significant at *p* < 0.05.

## 3. Results

### 3.1. Extraction Yield and Chromatographic Profiling of Phenolic Compounds in Cannabis sativa L. Seed Extract

**Figure 1:**
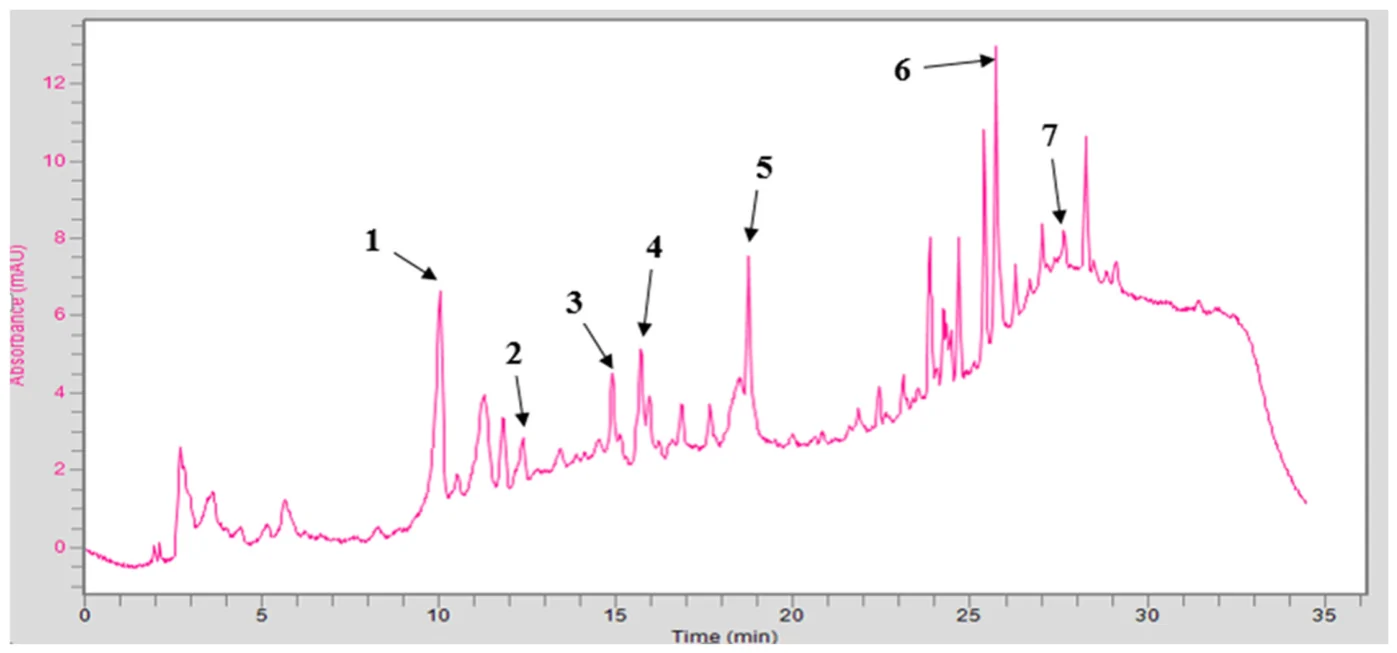
Chromatogram of hydroethanolic extract of *C. sativa* L. seed.

Under the experimental conditions, the hydroethanolic extraction of *C. sativa* seeds produced a yield of 23.80% (*w*/*w*). HPLC-DAD analysis of the *C. sativa* L. seed extract (CSSE) allowed the identification of seven major phenolic compounds, based on comparison of their retention times and DAD spectra with those of authentic standards (Figure 1, Table 1). The chromatographic profile revealed a clear predominance of catechin, which constituted the major compound of the extract with a relative peak area of 18.90%, followed by quercetin (9.89%) and vanillin (8.53%). *p*-Coumaric acid (6.37%) and caffeic acid (3.81%) were detected in intermediate proportions, while chlorogenic acid (1.5%) and kaempferol (1.32%) appeared as minor constituents of the extract. Altogether, these seven identified compounds accounted for approximately 50.3% of the total chromatographic area.

### 3.2. Evidence of Single-Dose Innocuity

**Figure 2:**
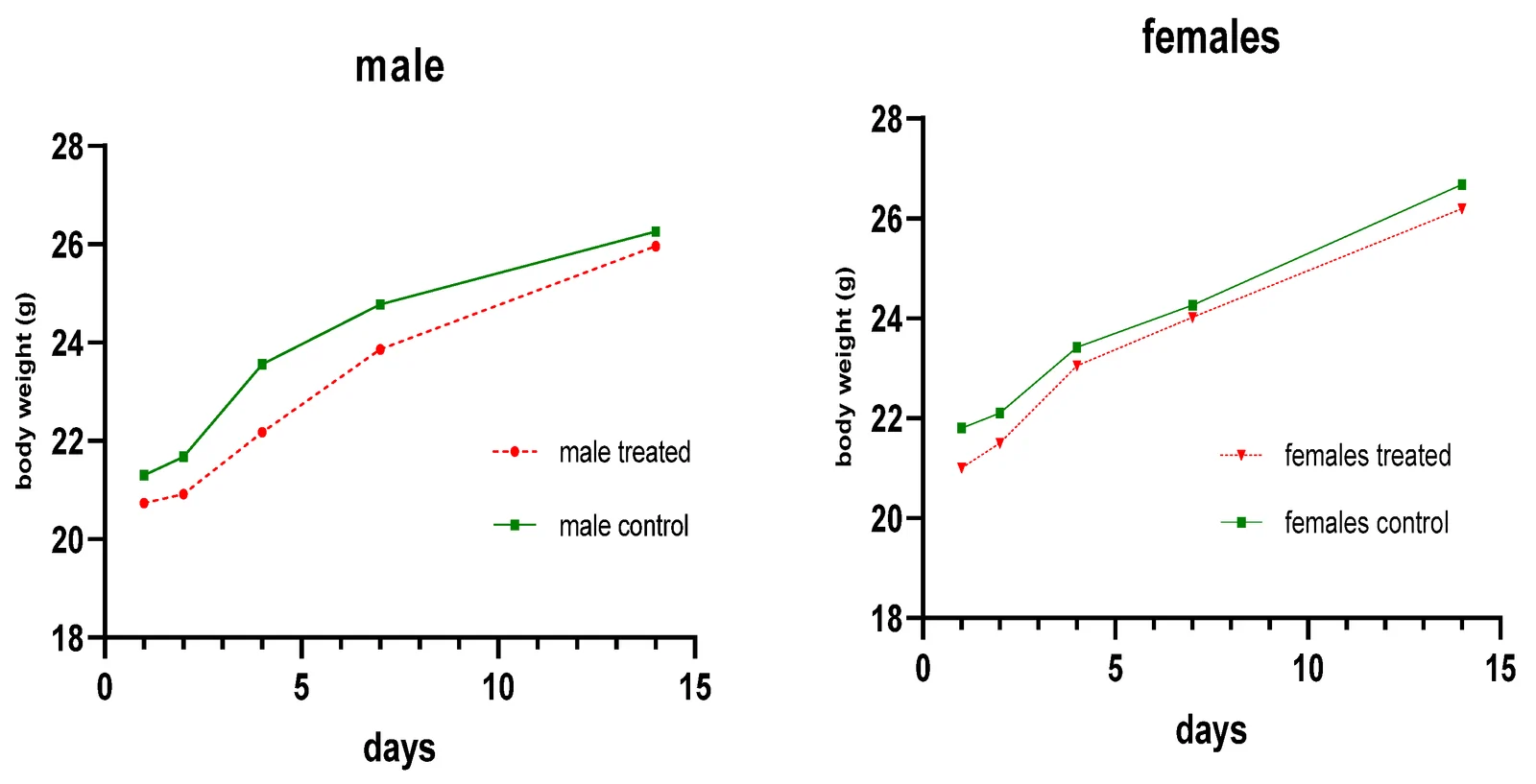
Effect of CSSE on body weight of mice during acute toxicity study.

During acute toxicity testing, animals were closely observed for signs of toxicity at 30 min, 1, 2, 4, and 8 h post-administration, and once daily thereafter for 14 days, in accordance with OECD guideline 423. No clinical signs of toxicity—including aggression, agitation, drowsiness, respiratory distress, seizures, hypoactivity, or paralysis—were observed in either the CSSE-treated group (5000 mg/kg) or the control group (distilled water, 1 mL/100 g), and no mortality occurred; all animals survived to the end of the observation period (Table S1). Body weight did not differ significantly between the two groups (*p* > 0.05) (Figure 2). Taken together, these findings indicate that CSSE produced no observable acute toxic effects at the limit dose of 5000 mg/kg, supporting its classification as practically non-toxic following single-dose oral administration in mice, consistent with OECD guideline 423.

#### 3.2.1. Sub-Acute Oral Toxicological Outcomes of *C. sativa* L. Seed Extract in Mice

##### Assessment of General Toxicity, Body Weight Dynamics, and Food-Water Consumption

**Figure 3:**
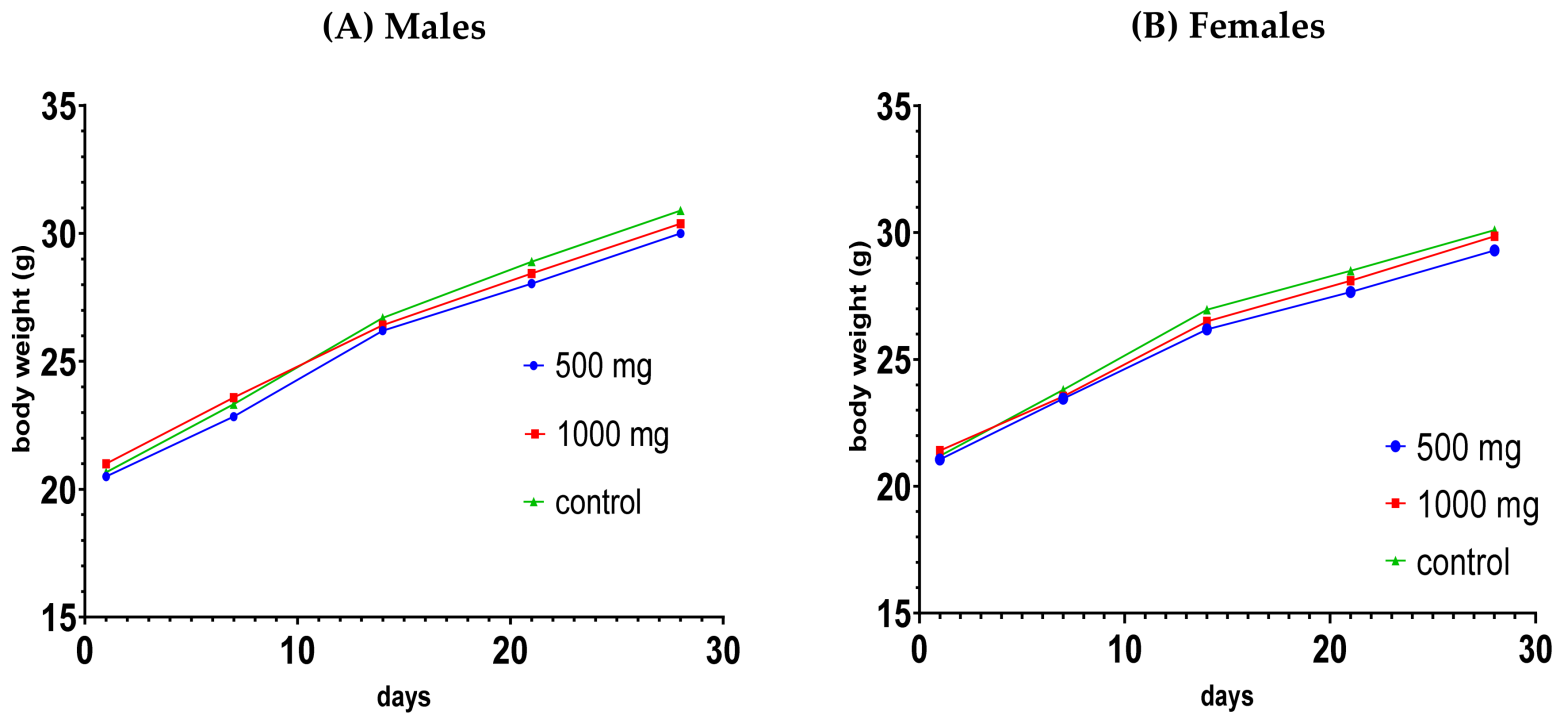
Effect of CSSE on body weight of mice during sub-acute toxicity study.

During the 28-day sub-acute toxicity investigation of *C. sativa* seed extract (CSSE) at doses of 500 and 1000 mg/kg b.w., no treatment-related mortality or alterations in behavioral patterns were observed in either male or female mice. Regarding body weight, differences between the treated and control groups were recorded and summarized in Figure 3; a progressive and statistically comparable weight gain was recorded across all experimental groups from day 7 through day 28 relative to baseline values on day 1 (*p* > 0.05). Specifically, the control group exhibited an initial mean body weight of 21.2 ± 0.74 and reached a final weight of 30.1 ± 0.53 g, while the 500 mg/kg group progressed from 21.06 ± 0.71 g to 29.3 ± 0.57 g, and the 1000 mg/kg group increased from 21.4 ± 0.96 g to 29.86 ± 0.40 g. These growth trajectories showed no statistically significant differences attributable to CSSE administration Figure 3. Furthermore, daily monitoring revealed that oral administration of *C. sativa* seed extract (CSSE) at doses of 500 and 1000 mg/kg produced no significant changes in food or water consumption across both male and female mice throughout the sub-acute treatment period (Table 2). In male mice, average daily food intake remained consistent between the control group (9.31 ± 0.86 g/day) and the groups treated with 500 mg/kg (9.64 ± 0.68 g/day) and 1000 mg/kg (9.58 ± 0.68 g/day). Similarly, daily water intake in males exhibited no meaningful deviation from baseline controls (15.4 ± 0.88 mL/day for control vs. 16.0 ± 0.40 mL/day and 16.3 ± 0.96 mL/day for the 500 and 1000 mg/kg groups, respectively). A similar pattern was recorded in female animals, where dietary intake remained comparable between non-treated controls (9.55 ± 0.75 g/day) and extract-treated groups (10.36 ± 1.31 g/day at 500 mg/kg and 10.50 ± 0.80 g/day at 1000 mg/kg). Water consumption in females likewise showed no treatment-related alterations, ranging from 17.2 ± 1.44 mL/day in controls to 17.8 ± 0.64 mL/day (500 mg/kg) and 17.4 ± 0.48 mL/day (1000 mg/kg).

Overall, these observations indicate that repeat-dose oral administration of CSSE did not disrupt metabolic demand, appetitive behavior, or hydration status in either sex.

#### 3.2.2. Variations in Hematological, Biochemical, and Hepatic Biomarkers Following *Cannabis sativa* L. Seed Extract Administration

The examination of blood parameters such as WBC, RBC, LYM, MONO, NEU, EOS, BAS, PLT, HGB, HCT, MCV, MCH, and MCHC in mice given CSSE at varying doses (500, 1000 mg/kg) showed no significant differences compared to the control group in both sexes. Nevertheless, when administered at the highest dose in female mice (1000 mg/kg), CSSE induced a significant elevation in platelet count (PLT) (*p* < 0.05) in the female group (Table 3).

The data on blood biochemistry are outlined in Table 4. Oral administration of CSSE significantly reduced serum total cholesterol in both sexes and triglycerides in females compared to the control group. In females, total cholesterol levels decreased significantly at 500 mg/kg/day (*p* < 0.01) and 1000 mg/kg/day (*p* < 0.001), while triglyceride levels were significantly reduced at the 1000 mg/kg/day dose (*p* < 0.05). In males, total cholesterol levels were significantly lowered at both 500 mg/kg/day (*p* < 0.05) and 1000 mg/kg/day (*p* < 0.01) relative to control.

#### 3.2.3. Morphological and Histological Outcomes: Necropsy Findings, Organ Masses, and Tissue Pathology

**Figure 4:**
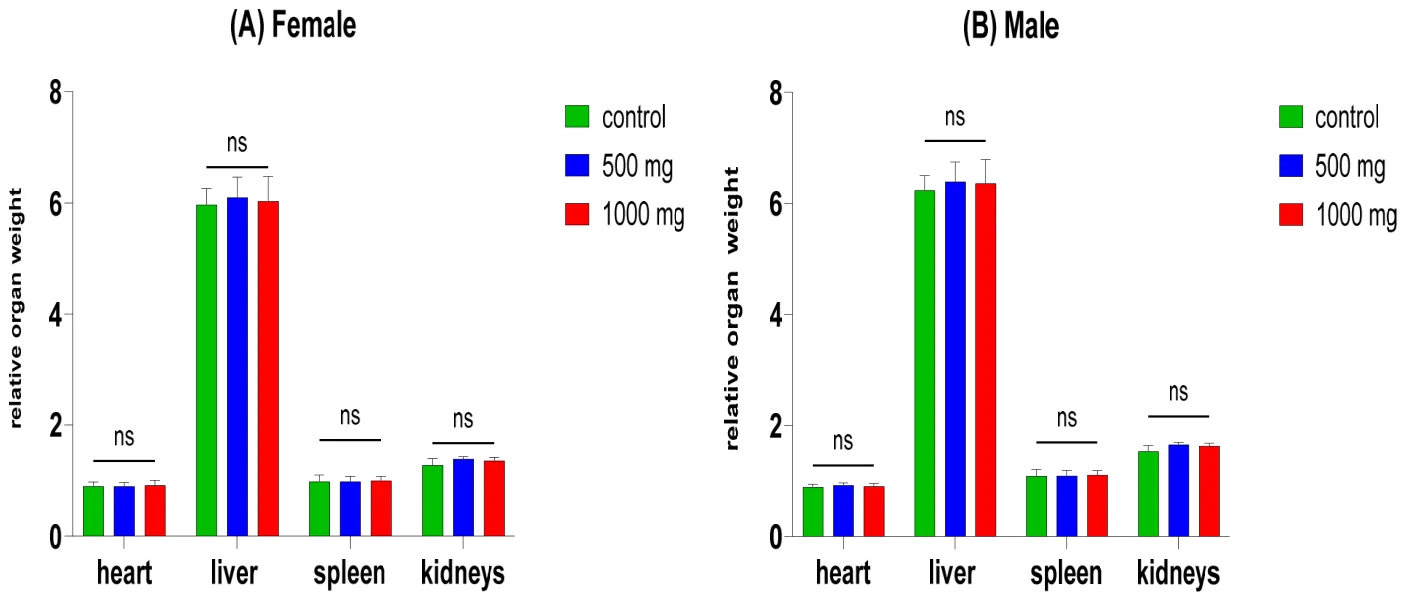
Mean relative organ weight (%) of vital organs after 28 days at different doses of CSSE (*n* = 5). Note: ns = no significant changes compared to the control group of mice (*p* > 0.05).

Macroscopic post-mortem inspection of the primary vital organs (including the liver, kidneys, heart, and spleen) revealed normal anatomical architecture without any visible signs of swelling, hyperemia, inflammation, hemorrhage, or localized tissue lesions across all experimental groups. Quantitative analysis of relative organ weights further corroborated these macroscopic observations. As illustrated in Figure 4, repeat-dose oral administration of *C. sativa* seed extract (CSSE) at 500 mg/kg and 1000 mg/kg over 28 consecutive days induced no statistically significant differences (*p* > 0.05) in relative organ mass compared to non-treated control animals. This absence of relative organ weight alterations was consistently observed in both male and female mice, indicating a lack of overt organ-specific toxicity or tissue hypertrophy.

**Figure 5:**
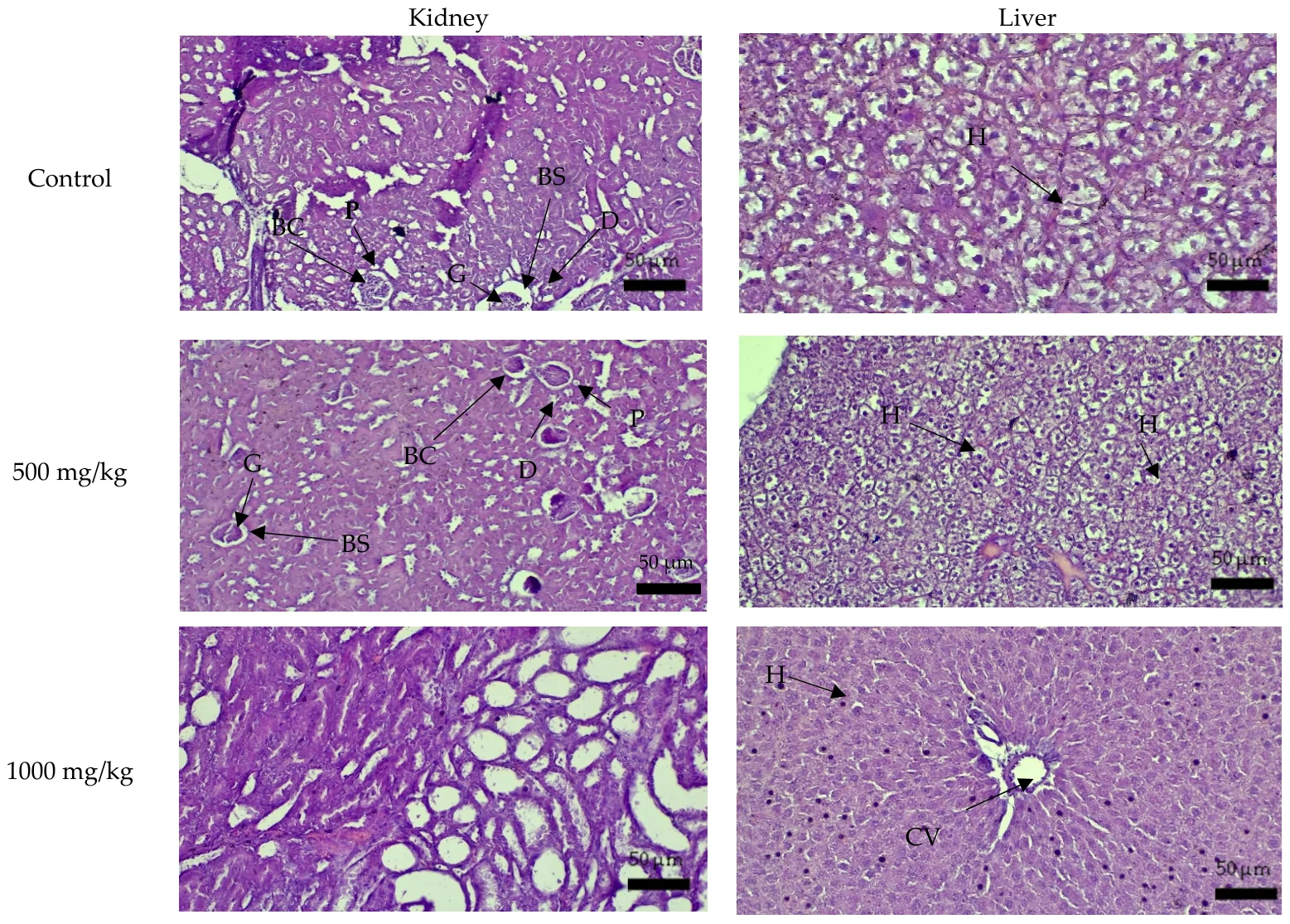
Histological findings from two organs (kidneys and liver) in mice following oral administration over a 28-day period (original magnification × 100). Note: BC: Bowman’s capsule; CV: central vein; H: hepatocytes; G: Glomerulus; BS: Bowman’s space; D: distal convoluted tubule; P: proximal convoluted tubules.

Histopathological examination of microsectioned tissues confirmed the organ weight and macroscopic findings (Figure 5). Microscopic evaluation focused on the two primary organs of metabolism and excretion, the liver and kidneys: Photomicrographs of liver sections from control and CSSE-treated mice (500 and 1000 mg/kg) demonstrated well-preserved hepatic lobular architecture. Hepatocytes were normally arranged in radial cords surrounding the central vein, displaying distinct round nuclei, intact cell boundaries, and homogeneous cytoplasm. No evidence of hepatocellular swelling, fatty degeneration (steatosis), portal inflammation, focal necrosis, or sinusoidal congestion was detected at any dose level. Microscopic sections of the kidneys exhibited intact structural integrity in both the cortical and medullary regions across all groups. Glomeruli showed normal cellularity and well-defined Bowman’s spaces. Renal convoluted tubules displayed intact epithelial linings without brush border loss, tubular lumen dilation, hyaline casts, or interstitial inflammatory infiltrates.

Overall, these structural and microscopic observations demonstrate that sub-acute exposure to CSSE at doses up to 1000 mg/kg does not induce structural toxicity, organomegaly, or tissue pathology in Swiss mice.

## 4. Discussion

*C. sativa* L. seeds used in Moroccan folk medicine [15] are a rich source of nutrients such as vitamins, dietary fiber, minerals, polyunsaturated fatty acids, essential fatty acids, and amino acids constituting easily digestible protein [16]. Similarly, cannabis seeds contain high amounts of polyphenols coupled with health benefits [17]. However, thus far, there have been no documented assessments of its toxicity. With a noticeable lack of literature on clinical trials, one can predict an overzealous approach to supra-supplementation, fueled by the misguided belief that “more is better”. Therefore, conducting advanced oral toxicity research is crucial for determining doses that could be utilized in the future and identifying potential clinical signs induced by *C. sativa* L. seed consumption; additionally, it can help establish its therapeutic potential.

The importance of using animal studies to predict toxic effects in humans has been confirmed to have a high predictive value [18]. To precisely evaluate potential toxicity, both acute and sub-acute toxicity evaluations were conducted using male and female mice; the inclusion of both genders in drug research is attributed to the significant variations in drug reactions between male and female bodies [19,20]. Furthermore, the interaction between estrogen and various enzymes or receptors can either diminish or amplify drug effects, potentially impacting toxic side effects [21].

Acute oral toxicity refers to adverse effects occurring shortly after the administration of a test substance, typically within 24 h [22]. In the current study, administering a high dose of 5000 mg/kg body weight of hydro-ethanolic extract from *C. sativa* L. seeds did not induce toxic reactions, cause mortality, or alter body weight. This finding suggests that the lethal dose 50 (LD50) could potentially exceed 5000 mg/kg. Similarly, a study by El-Mernissi et al. [23] found that acute exposure to *C. sativa* L. essential oil at a dose of 2 g/kg body weight did not result in mortality or adverse effects in male and female mice after 24 h of treatment [23]. More recently, El Mskini et al. (2026) specifically investigated the acute toxicity of a chloroform extract prepared from *C. sativa* L. seeds in female Swiss mice according to OECD Guideline 425 [24]. A single oral dose of 2000 mg/kg resulted in no treatment-related mortality during the 14-day observation period, corresponding to an LD50 > 2000 mg/kg. Although the extraction solvent and dose differed from those used in the present study, these findings provide directly relevant evidence regarding the acute safety profile of *C. sativa* seed extracts. In contrast, Balafrej et al. (2023) evaluated extracts obtained from female-plant co-products of *C. sativa* using maceration, decoction, and sonication procedures at 2000 mg/kg and reported varying intensities of toxicity-related signs [25]. These differences highlight the importance of considering the specific plant part, extraction solvent, and preparation method when comparing toxicological findings among *C. sativa* studies.

Changes in body weight are important indicators of potential adverse effects in repeated-dose toxicity studies, as marked changes may reflect systemic toxicity [26], Food and water consumption are also relevant parameters for evaluating possible effects on appetite and general physiological status [27]. In the present sub-acute study, both control and treated groups showed a progressive increase in body weight, with no significant differences in food or water intake, suggesting that the hydro-ethanolic seed extract did not adversely affect these parameters under the experimental conditions [28]. Consistent with these observations, El Mskini et al. (2026) reported no significant difference in body-weight gain following acute oral administration of a chloroform extract specifically prepared from *C. sativa* L. seeds at 2000 mg/kg in female Swiss mice [24]. Similar results were observed by Bailey, who investigated a water-soluble cannabinol preparation [29].

Organ weight is a crucial indicator of an animal’s physiological and pathological status. As an organism grows, the weight of its individual organs will also increase, making the organ-to-body weight ratio (relative organ index) predictable; deviations from this expected index can indicate organ enlargement (such as from congestion or edema) or a decrease in organ size (due to necrosis or atrophy), both of which can be associated with toxicity [30]. In this investigation, visual inspection of the internal organs in all mice showed no detectable abnormalities. Additionally, administering the extract at both doses did not cause significant changes in the weights of the various vital organs compared with the control group in both sexes. These outcomes align with the findings of earlier studies [25,29,31].

The hematological system serves as a critical indicator of physiological and pathological conditions in animals [32]. In the present study, most hematological parameters remained comparable between treated and control groups in both sexes. However, a significant decrease in platelet count was observed exclusively in female mice receiving 1000 mg/kg body weight (*p* < 0.05). This sex-specific response may potentially reflect differences between males and females in the pharmacokinetics, metabolism, or biological response to plant-derived constituents. Sex hormones and sex-dependent differences in hepatic biotransformation and systemic exposure may also influence susceptibility to certain xenobiotics [33,34,35]. Nevertheless, the present study did not include specific investigations of bone marrow function, platelet production, or platelet turnover; therefore, a direct mechanism for the observed decrease cannot be established. Moreover, the isolated nature of this finding, in the absence of consistent alterations in the other hematological parameters or histopathological abnormalities, warrants cautious interpretation. Further sex-specific investigations would be necessary to determine whether this finding represents a biologically relevant treatment-related effect.

It is highly sensitive to toxic substances, making even slight changes in hematological parameters valuable for detecting drug-induced toxicity, often providing early signs of adverse effects [36]. In our study, aside from platelets, hematological parameters in animals from various treated groups, regardless of sex, were comparable to those in corresponding control groups. Sub-acute exposure of female mice to 1000 mg/kg body weight resulted in a notable decrease in platelet count (*p* < 0.05). This decrease may be attributed to factors such as direct toxicity affecting the bone marrow, where platelets are produced, or disruptions in their normal function and lifespan, leading to premature destruction or impaired activity. It is crucial to emphasize that a reduced platelet count can have serious implications, as platelets are essential for effective blood clotting. This condition, known as thrombocytopenia, can lead to significant bleeding complications [37].

Clinical biochemistry primarily aims to assess the effects of drugs on liver and kidney functions, as well as their influence on total cholesterol and glucose levels [32]. Given that the liver is the primary organ responsible for the drug biotransformation process, toxicity investigation must include a comprehensive evaluation of liver function and structure. Key biochemical markers used to assess liver damage and functionality encompass total protein and specific liver enzymes such as ALT and AST. Additionally, because the liver plays a crucial role in glucose production and cholesterol metabolism, fasting blood glucose (FBG), cholesterol total (CT), and triglycerides (TG) can be used as indirect indicators of liver function [38]. The present study showed no significant differences in the aforementioned biochemical parameters between mice treated with the test substance at any dose in both genders and the control groups, except for cholesterol total (CT) and triglycerides (TG), which showed a significant diminution at a dose of 1000 mg/kg (*p* < 0.01, *p* < 0.05), respectively.

Similar to the liver, the kidneys are also frequently susceptible to damage from toxic substances [39]. In this study, creatinine and urea determinations were critical as markers of kidney function [40,41], the groups fed with *C. sativa* L. seed extract exhibited negligible differences in urea and creatinine levels, suggesting that *C. sativa* L. seeds might not affect normal renal function following sub-acute exposure compared to the control groups.

Evaluating histopathological changes in organs is a fundamental part of drug safety assessment [42]. Histological analysis of liver and kidney tissues showed no notable damage or alterations, which aligns with findings from macroscopic observation and biochemical parameter evaluations. In contrast, Yassa et al. (2010) investigated the subchronic toxicity of *C. sativa* L. leaves (“bango”) in male albino rats following repeated exposure for 90 days and reported histopathological alterations in several organs [31].

This study offers valuable insights into the acute and sub-acute toxicity profile of hydro-ethanolic extracts of *C. sativa* L. seeds, which will benefit future in vivo and clinical research on this plant medicine. The extract of *C. sativa* L. seeds exhibited non-toxic effects when subjected to oral sub-acute toxicity tests in mice. These findings suggest that *C. sativa* L. seed extract is safe, supporting its widespread use in folk medicine and nutrition.

## 5. Conclusions

In the present study, we investigated the acute and sub-chronic toxicities of *C. sativa* L. seed hydro-ethanolic extracts by oral administration in mice. Acute toxicity results showed that a single oral dose of 5000 mg/kg did not cause mortality or sudden death, indicating that the LD_50_ in mice exceeds 5000 mg/kg and classifying the extract as practically non-toxic. The 28-day sub-chronic toxicity assessment revealed no toxicity at daily oral doses of 500 mg/kg and 1000 mg/kg, as determined by hematological, serum biochemical, and histological analyses. It could be deemed reliable for further medicinal applications. Additionally, chronic toxicity, carcinogenicity, and mutagenicity studies should be conducted to better understand the safety profile of *C. sativa* L. seeds.

## Figures and Tables

**Table 1:** Identified compounds by HPLC-DAD in *C. sativa* L. seed extract.

Peak Number	Compound	Retention Time (min)	Area %	Structure
1	Catechin	10.04	18.90	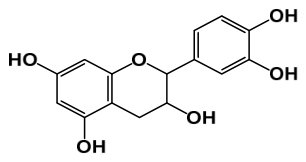
2	Chlorogenic Acid	12.05	1.5	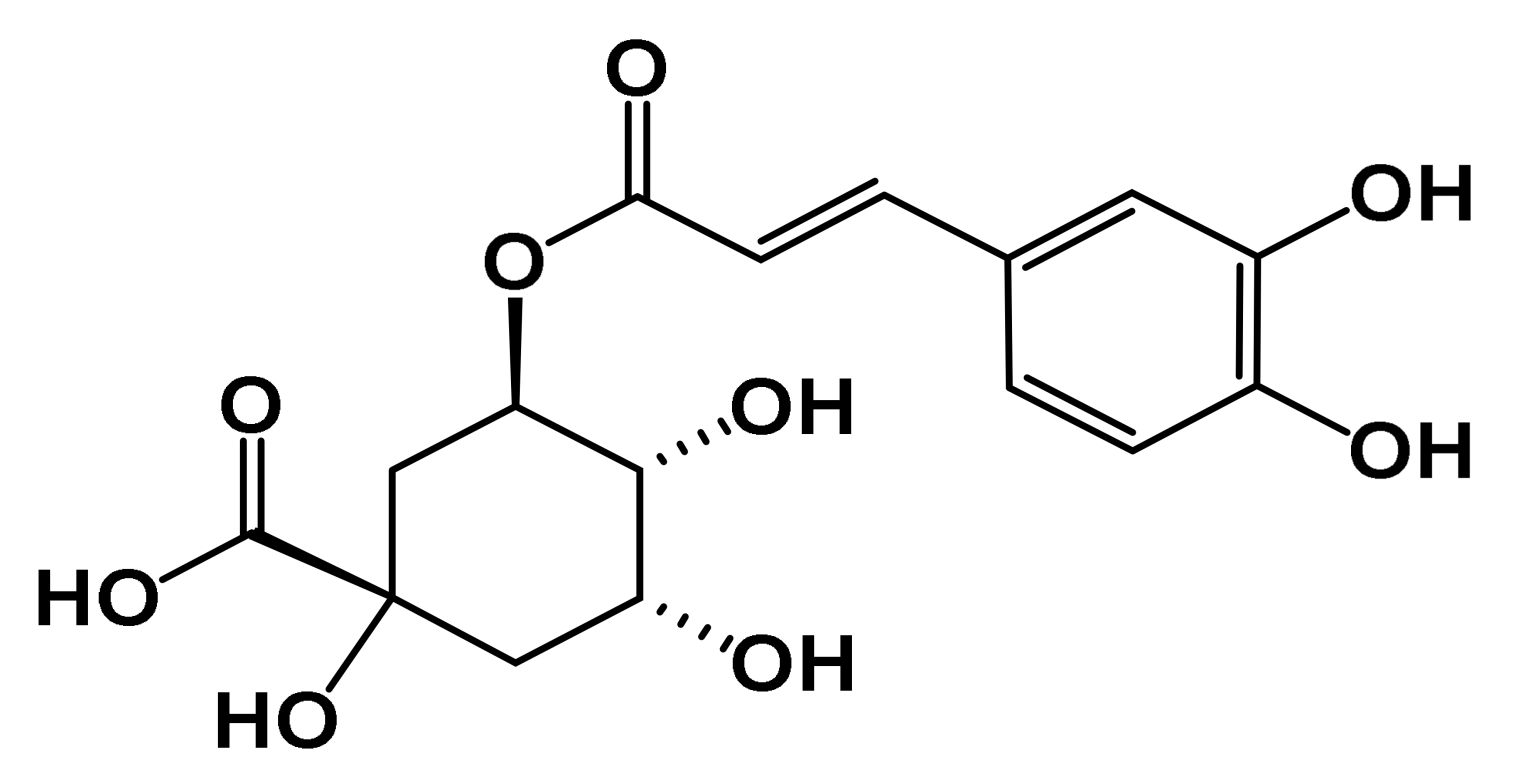
3	Caffeic acid	14.91	3.81	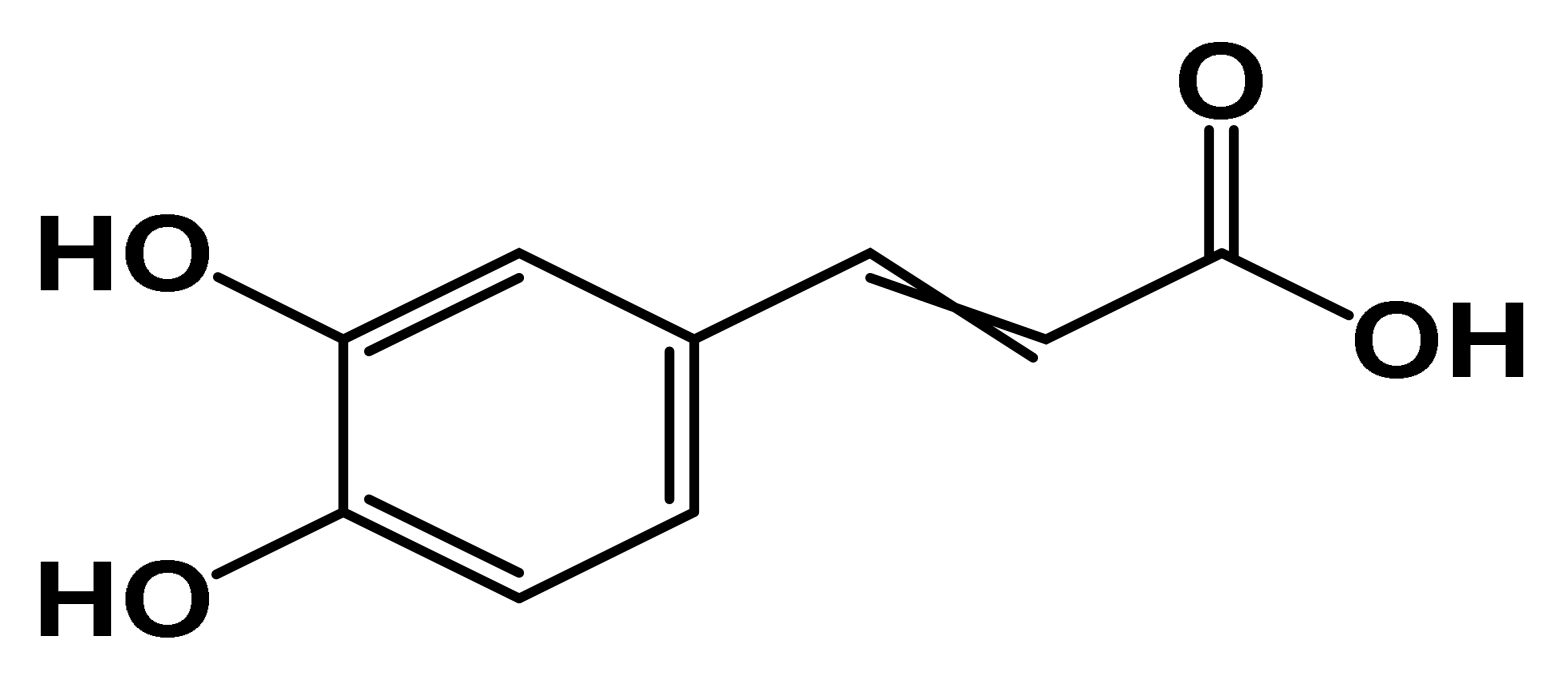
4	Vanillin	15.71	8.53	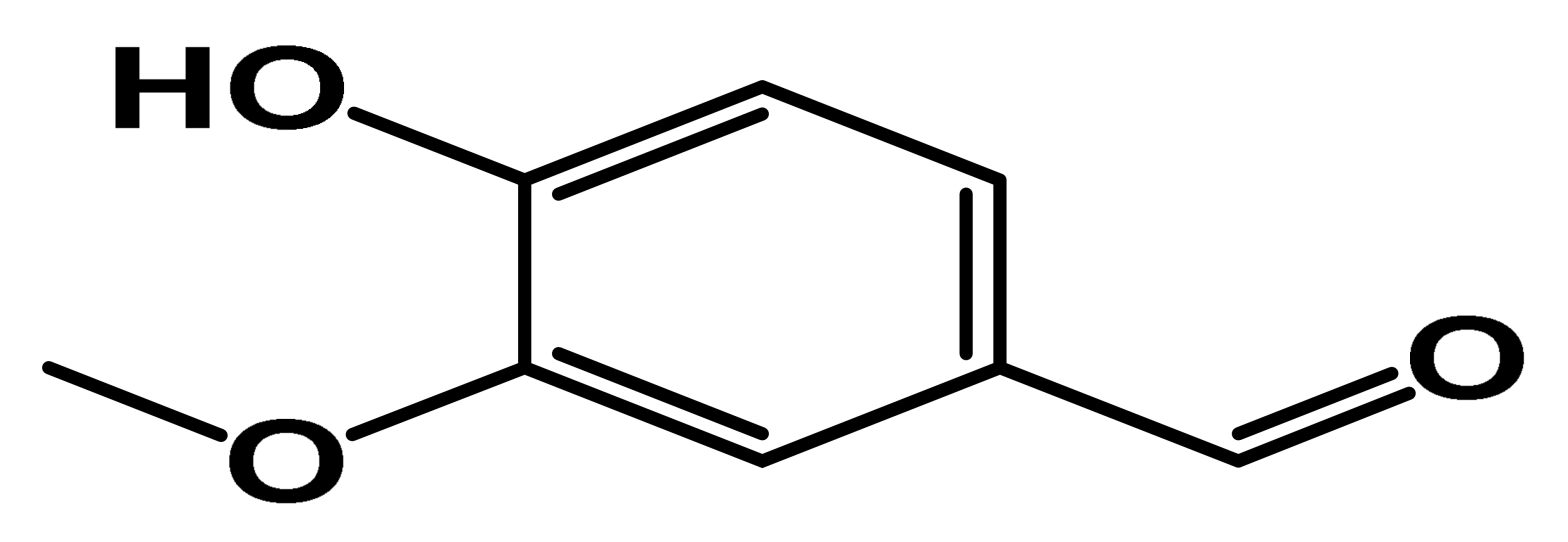
5	*p*-Coumaric acid	18.74	6.37	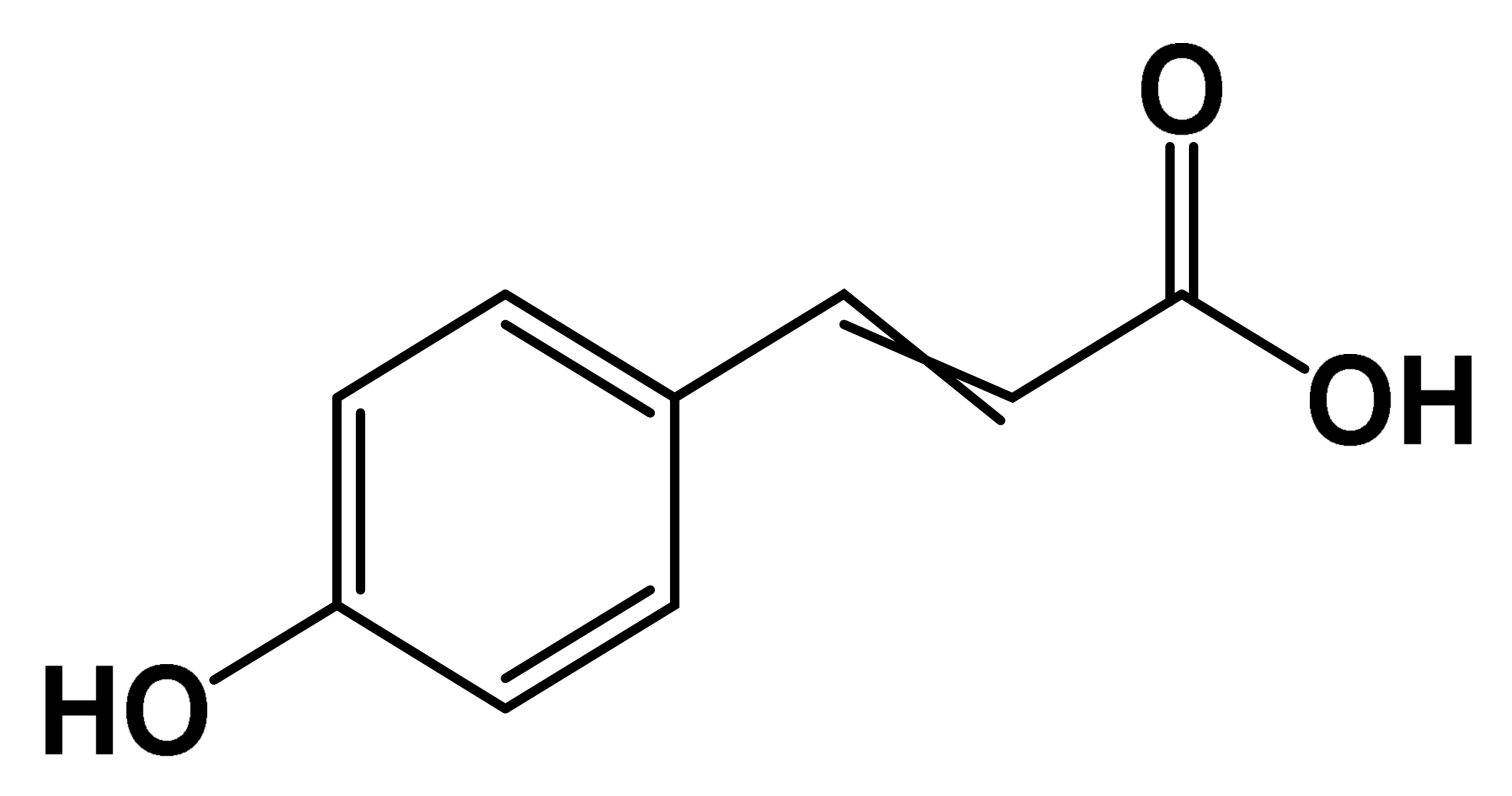
6	Quercetin	25.71	9.89	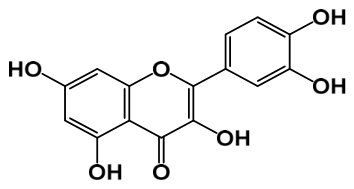
7	Kaempferol	27.63	1.32	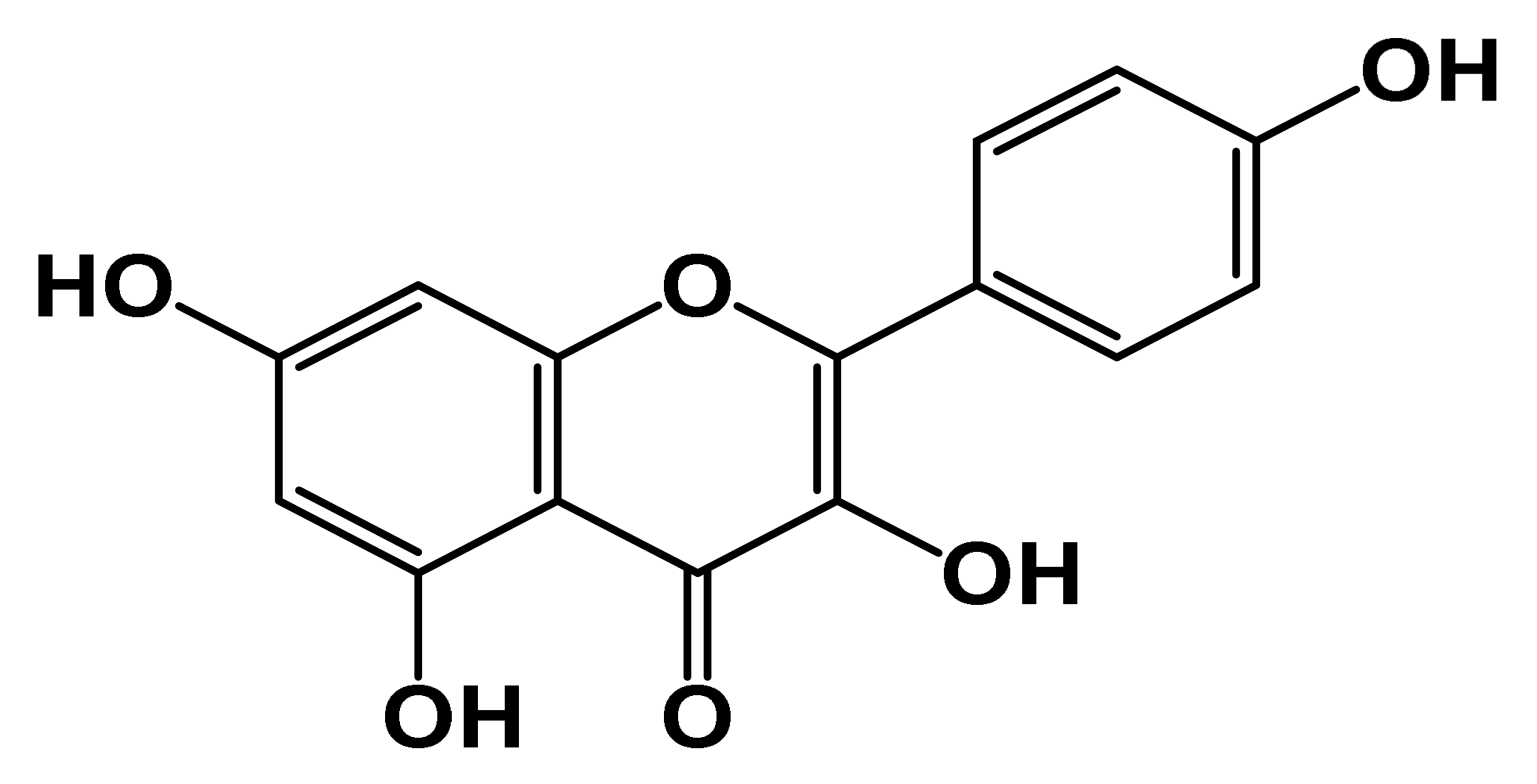

**Table 2:** Effect of CSSE on food intake and water intake in mice in a sub-acute toxicity study.

	Male		Female	
	Food Intake (g/Day/Group)	Water Intake (ml/Day/Group)	Food Intake (g/Day/Group)	Water Intake (ml/Day/Group)
control	9.31 ± 0.86	15.4 ± 0.88	9.55 ± 0.75	17.2 ± 1.44
500 mg	9.64 ± 0.68	16 ± 0.4	10.36 ± 1.312	17.8 ± 0.64
1000 mg	9.58 ± 0.68	16.3 ± 0.96	10.5 ± 0.8	17.4 ± 0.48

All values were presented as Mean ± SD (*n* = 5).

**Table 3:** The influence of sub-acute oral intake of *C. sativa* L. seeds hydroethanolic extract on hematological parameters in mice.

Sex	Hematological Parameter	Study Group		
		Control	500 mg	1000 mg
Male	White blood cell (WBC) (10^3^/mm^3^)	3.759 ± 0.085	3.75960 ± 0.047	3.7198 ± 0.029
	Red blood cell (RBC) (M/mm^3^)	8.53 ± 0.62	8.41 ± 0.26	8.718 ± 0.49
	Lymphocyte (LYM) (10^3^/mm^3^)	77.78 ± 1.27	79.03 ± 0.45	78.212 ± 0.92
	Monocyte (10^3^/mm^3^)	1.42 ± 0.35	2.34 ± 0.43	1.57 ± 0.39
	Neutrophil (NEU) (10^3^/mm^3^)	18.04 ± 1.19	15.36 ± 0.46	17.48 ± 0.84
	Eosinophils (EOS) (10^3^/mm^3^)	0.22 ± 0.05	0.77 ± 0.09	1.23 ± 0.18
	Basophils (BAS) (10^3^/mm^3^)	2.72 ± 0.19	2.52 ± 0.33	1.6 ± 0.28
	Platelet count (PLT) (10^3^/mm^3^)	847.40 ± 14.91	840.20 ± 23.86	837.4 ± 16.53
	Hemoglobin (HGB) (g/dL)	10.84 ± 0.88	11.60 ± 0.38	11.55 ± 0.80
	Hematocrit (HCT) (%)	44.80 ± 1.87	45.02 ± 1.44	42.51 ± 1.38
	Mean corpuscular volume (MCV) (μ^3^)	49.48 ± 1.60	48.60 ± 3.05	49.23 ± 1.53
	Mean corpuscular hemoglobin (MCH) (pg)	12.66 ± 1.00	12.46 ± 1.16	12.42 ± 0.43
	Mean corpuscular hemoglobin concentration (MCHC) (g/dL)	25.34 ± 2.02	24.18 ± 1.76	25.9 ± 0.74
Female	White blood cell (WBC) (10^3^/mm^3^)	3.741 ± 29	3.761 ± 0.36	3.7858 ± 0.34
	Red blood cell (RBC) (M/mm^3^)	9.09 ± 0.14	9.18 ± 0.26	9.44 ± 0.224
	Lymphocyte (LYM) (10^3^/mm^3^)	78.81 ± 0.90	77.62 ± 1.14	76.39 ± 0.864
	Monocyte (10^3^/mm^3^)	1.70 ± 0.20	1.90 ± 0.16	1.466 ± 0.3128
	Neutrophil (NEU) (10^3^/mm^3^)	16.94 ± 0.75	17.32 ± 0.84	18.98 ± 0.424
	Eosinophils (EOS) (10^3^/mm^3^)	0.29 ± 0.03	0.29 ± 0.04	0.318 ± 0.0144
	Basophils (BAS) (10^3^/mm^3^)	2.64 ± 0.31	2.67 ± 0.30	2.78 ± 0.264
	Platelet count (PLT) (10^3^/mm^3^)	962.80 ± 20.64	959 ± 23.99	904 ± 18.48 *
	Hemoglobin (HGB) (g/dL)	11.58 ± 0.78	11.71 ± 0.47	11 ± 0.8
	Hematocrit (HCT) (%)	43.09 ± 2.72	41.20 ± 2.77	43.6 ± 3.68
	Mean corpuscular volume (MCV) (μ^3^)	53.35 ± 0.78	52.80 ± 2.28	53.2 ± 2.56
	Mean corpuscular hemoglobin (MCH) (pg)	15.52 ± 0.67	14.95 ± 0.44	16.14 ± 0.688
	Mean corpuscular hemoglobin concentration (MCHC) (g/dL)	26.93 ± 0.91	27.20 ± 1.30	30.6 ± 3.28

Results are expressed as mean ± SD (*n* = 5). * Significantly different from the control group, *p* < 0.05.

**Table 4:** The influence of sub-acute oral intake of *C. sativa* L. seeds hydroethanolic extract on biochemical parameters in mice.

Sex	Biochemical Parameter	Study Group		
		Control	500 mg	1000 mg
Male	Fasting blood glucose (FBG) mmol/L	10.10 ± 0.60	9.84 ± 0.54	11.00 ± 0.71
	Urea (Ur) mmol/L	11.02 ± 0.81	9.64 ± 0.66	10.29 ± 0.36
	Creatinine μmol/L	28.44 ± 0.93	29.38 ± 1.79	30.02 ± 1.01
	Total protein (TP) g/L	57.80 ± 0.84	58.00 ± 1.58	59.20 ± 1.92
	Total cholesterol (TC) mmol/L	2.34 ± 0.15	2.11 ± 0.15 *	2.06 ± 0.16 **
	TRIGLYCERIDE mmol/L	1.57 ± 0.10	1.4 ± 0.06	1.37 ± 0.12 *
	Aspartate aminotransferase AST (TGO) U/L	259.20 ± 6.06	260.40 ± 3.21	258.00 ± 3.08
	Alanine aminotransferase ALT (GPT) U/L	60.60 ± 2.41	59.20 ± 1.64	58.40 ± 1.14
Female	Fasting blood glucose (FBG) mmol/L	9.88 ± 0.31	10.35 ± 0.75	10.1 ± 0.36
	Urea (Ur) mmol/L	9.44 ± 0.35	10.14 ± 0.74	10.26 ± 0.392
	Creatinine μmol/L	26.70 ± 0.64	27.68 ± 1.19	29.2 ± 1.04
	Total protein (TP) g/L	61.00 ± 1.20	58.00 ± 1.58	57.08 ± 0.368
	Total cholesterol (TC) mmol/L	2.54 ± 0.19	2.19 ± 0.18 **	2.08 ± 0.06 ***
	Triglyceride mmol/L	1.66 ± 0.27	1.40 ± 0.08	1.36 ± 0.11 *
	Aspartate aminotransferase AST (TGO) U/L	234.20 ± 3.04	232.00 ± 7.11	230.6 ± 4.96
	Alanine aminotransferase ALT (GPT) U/L	53.00 ± 1.60	50.60 ± 2.70	49.6 ± 1.52

Results are expressed as mean ± SD (*n* = 5). * Significantly different from the control group, *p* < 0.05. ** Significantly different from the control group, *p* < 0.01. *** Significantly different from the control group (*p* < 0.001).

## Data Availability

All data supporting the findings of this study are included in the article and its Supplementary Materials.

## Supplementary Materials

The following supporting information can be downloaded at: https://www.mdpi.com/article/10.3390/molecules31203587/s1, Figure S1: Sequential decision scheme of OECD Guideline 423 used for the acute oral toxicity assessment of *C. sativa* L. seed extract; Table S1: Acute oral toxicity observations following administration of CSSE at 5000 mg/kg body weight.
