# Supplementary material for: Phenolic Profiling and OECD-Guided Acute and Subacute Oral Toxicity Assessment of Cannabis sativa L. Seed Hydroethanolic Extract
Source: Molecules. 2026 Oct 9;31(20):3587. doi: 10.3390/molecules31203587 (PMC13707569; doi:10.3390/molecules31203587)
Supplement: Supplementary file 1 [file molecules-31-03587-s001.zip › molecules-4550559-supplementary.pdf]

molecules

MDPI

molecules-4550559 Supplementary Material

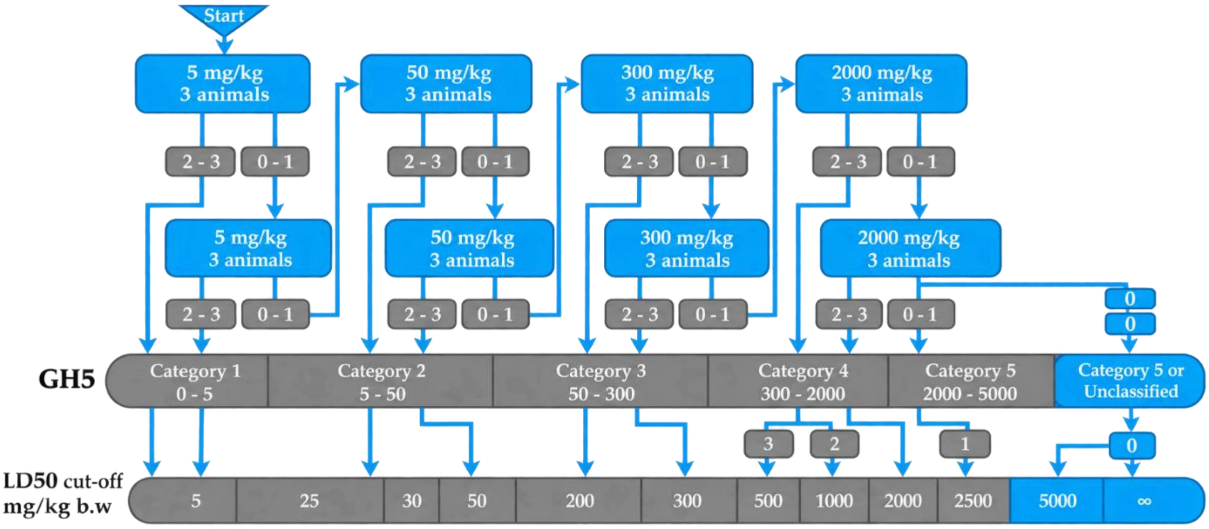

(Supplementary Figure S1: Sequential decision scheme of OECD Guideline 423 used for the acute oral toxicity assessment of Cannabis sativa L. seed extract.).

Table S1. Acute oral toxicity observations following administration of CSSE at 5000 mg/kg body weight.

**Observations**	G1 = CSSE (5000mg/kg)									G2= distilled water 1ml/100g							
**Behavioral pattern**	30 min	1 h	2 h	4h	8h	Day 1	Day 4	Day 7	Day 14	30 min	1h	2 h	4h	Day 1	Day 4	Day 7	Day 14
aggression	-	-	-	-	-	-	-	-	-	-	-	-	-	-	-	-	-
Agitation	-	-	-	-	-	-	-	-	-	-	-	-	-	-	-	-	-
drowsiness	-	-	-	-	-	-	-	-	-	-	-	-	-	-	-	-	-
breathing difficulties	-	-	-	-	-	-	-	-	-	-	-	-	-	-	-	-	-
seizures	-	-	-	-	-	-	-	-	-	-	-	-	-	-	-	-	-
hypoactivity	-	-	-	-	-	-	-	-	-	-	-	-	-	-	-	-	-
paralysis	-	-	-	-	-	-	-	-	-	-	-	-	-	-	-	-	-
Death	-	-	-	-	-	-	-	-	-	-	-	-	-	-	-	-	-
(-): No toxic symptoms or mortality were observed.																	
